# ATP Content and Cell Viability as Indicators for Cryostress Across the Diversity of Life

**DOI:** 10.3389/fphys.2018.00921

**Published:** 2018-07-17

**Authors:** Felizitas Bajerski, Johanna Stock, Benjamin Hanf, Tatyana Darienko, Elke Heine-Dobbernack, Maike Lorenz, Lisa Naujox, E. R. J. Keller, H. M. Schumacher, Thomas Friedl, Sonja Eberth, Hans-Peter Mock, Olaf Kniemeyer, Jörg Overmann

**Affiliations:** ^1^Leibniz Institute DSMZ - German Collection of Microorganisms and Cell Cultures, Braunschweig, Germany; ^2^Leibniz Institute of Plant Genetics and Crop Plant Research (IPK), Gatersleben, Germany; ^3^Leibniz Institute for Natural Product Research and Infection Biology e.V. - Hans-Knöll-Institute (HKI), Jena, Germany; ^4^Institute of Microbiology, Friedrich Schiller University Jena, Jena, Germany; ^5^Experimental Phycology and Culture Collection of Algae, University of Göttingen (EPSAG), Göttingen, Germany

**Keywords:** cryopreservation, viability tests, ultradeep freezing, ATP level, adaptation, physiological, cold stress, cold temperature

## Abstract

In many natural environments, organisms get exposed to low temperature and/or to strong temperature shifts. Also, standard preservation protocols for live cells or tissues involve ultradeep freezing in or above liquid nitrogen (-196°C or -150°C, respectively). To which extent these conditions cause cold- or cryostress has rarely been investigated systematically. Using ATP content as an indicator of the physiological state of cells, we found that representatives of bacteria, fungi, algae, plant tissue, as well as plant and human cell lines exhibited similar responses during freezing and thawing. Compared to optimum growth conditions, the cellular ATP content of most model organisms decreased significantly upon treatment with cryoprotectant and cooling to up to -196°C. After thawing and a longer period of regeneration, the initial ATP content was restored or even exceeded the initial ATP levels. To assess the implications of cellular ATP concentration for the physiology of cryostress, cell viability was determined in parallel using independent approaches. A significantly positive correlation of ATP content and viability was detected only in the cryosensitive algae *Chlamydomonas reinhardtii* SAG 11-32b and *Chlorella variabilis* NC64A, and in plant cell lines of *Solanum tuberosum*. When comparing mesophilic with psychrophilic bacteria of the same genera, and cryosensitive with cryotolerant algae, ATP levels of actively growing cells were generally higher in the psychrophilic and cryotolerant representatives. During exposure to ultralow temperatures, however, psychrophilic and cryotolerant species showed a decline in ATP content similar to their mesophilic or cryosensitive counterparts. Nevertheless, psychrophilic and cryotolerant species attained better culturability after freezing. Cellular ATP concentrations and viability measurements thus monitor different features of live cells during their exposure to ultralow temperatures and cryostress.

## Introduction

Over 80% of terrestrial and marine habitats are considered as cold, with temperatures residing permanently ≤15°C (Kirby et al., 2012). Cold stress of an organism occurs when outside temperatures fall below the optimum temperature range for growth and involves a slow-down of biochemical reactions, a decrease in pH of biological buffers, reduced membrane fluidity, cold denaturation of proteins, and a hydration of non-polar protein groups, affecting protein solubility and stability (Deming, 2002; Georlette et al., 2004). At temperatures below the freezing point, such as in arctic and antarctic environments, glacial and lake ice, high-altitude alpine sites, but temporarily also occurring in temperate regions (Georlette et al., 2004; Layborn-Parry et al., 2012; De Maayer et al., 2014), cells undergo cryostress, elicited by the formation of ice crystals. At slow cooling rates, ice formation often starts in the extracellular space whereas rapid cooling promotes intracellular crystallization (Mazur, 1969; Wisniewski and Fuller, 1999; Mazur, 2004). The resulting extracellular or intracellular decrease in water activity causes osmotic water efflux or influx, respectively, that effects the survival of the cells (Dumont et al., 2004). In addition, thawing or warming of cells may lead to recrystallization and the growth of larger ice crystals at the expense of smaller ones, which can cause significant cellular damage (Mazur, 1977).

Cellular adaptations to temperature decline involve the differentiation of permanent cell forms such as spores or akinetes, changing the phospholipid fatty acid inventory to maintain the functionality of cellular membranes (Bajerski et al., 2017), increasing the concentrations of enzymes (Willem et al., 1999), expressing cold-adapted isoenzymes (Hoyoux et al., 2001) or cold shock proteins stabilizing different cell constituents or changing gene expression (Phadtare et al., 1999; Weber and Marahiel, 2003; Wilson and Nierhaus, 2004), or preventing intracellular ice nucleation through the accumulation of sugars, or cryoprotectants like polyols (glycerol, arabitol, trehalose, and mannitol), secondary metabolites, or anti-freezing proteins (Duman and Olsen, 1993; Davies and Sykes, 1997; Sdebottom et al., 1997; Grant, 2004; Agrawal, 2009; De Maayer et al., 2014). Ice crystal formation can be inhibited and cellular proteins and membranes maintained in their native structure through the addition of glycerol or artificial cryoprotectants, particularly dimethyl sulfoxide (DMSO; Mazur, 1984). However, the physiological details of responses to rapid temperature decrease have mostly been studied at non-freezing temperatures [(cold stress; Graumann and Marahiel, 1996; Phadtare, 2004; Hannah et al., 2005; Cavicchioli, 2006), among many others] rather than under conditions of freezing (cryostress; Dumont et al., 2004; Miladi et al., 2013; Mykytczuk et al., 2013).

The different responses of living cells to cryostress are not only of fundamental scientific interest, but have direct practical significance for cryopreservation. Various bacteria and algae do not survive freezing under standard laboratory conditions (Smith, 2001; Day and Stacey, 2007). Standard preservation protocols for live cells or tissues involve ultradeep freezing in or above liquid nitrogen (-196°C or -150°C, respectively; Jacobsen and Stewart, 1973; Talwar, 2012). Therefore, an efficient cryopreservation is of increasing importance to safeguard biomaterials for follow-up scientific investigations (Schüngel et al., 2014), for subsequent medical applications, and for the maintenance of genetic resources in the agricultural sector, e.g., of crop plants which cannot be stored as seeds.

In order to monitor the physiological consequences of cryostress and to establish cryopreservation conditions, a suitable indicator for the physiological state of cells is needed. Across different cell types and tissues, the cellular content of ATP of growing cells is tightly regulated and maintained within a narrow concentration range (Holm-Hansen, 1970; Atkinson, 1977). Total cellular ATP content was suggested as a viability marker for cryopreserved cells, tissues and organs (Pegg, 1989) and cell cultures in general (Crouch et al., 1993; Niles et al., 2009). Cellular ATP concentration has been used in the past to monitor the physiological state of diverse prokaryotic and eukaryotic cells under starvation and (cold) stresses (e.g., Sobczyk et al., 1985; De Baulny et al., 1999; Napolitano and Shain, 2005; Amato and Christner, 2009; Tsai et al., 2010; Lin et al., 2011). Enzymes which are directly (F1-ATPase, V-ATPase) or indirectly related to adenylate anabolism might not work efficiently at subzero temperature. For example, a V-type ATPase is inhibited under cold stress in plants (Dietz et al., 2001). As a result the ATP level decreases (Napolitano and Shain, 2005). However, these enzymes differ across the diversity of life. Moreover, the kinases and phosphatases involved in the signal transduction during stress response affect intracellular adenylate levels (Corton et al., 1994; Huang et al., 2012). Therefore, ATP is a suitable biomarker to address cellular stress response. Indeed, cell damage induced by low temperatures has been linked to the shortage of cellular ATP (Amato and Christner, 2009).

In the present comparative study, a wide range of different cell types and tissues were subjected to cryostress to determine which parameters are suitable for monitoring the individual responses during freezing at ultralow temperatures as it occurs during cryopreservation. We show that cells and organisms across the diversity of life forms display changes of their cellular energy metabolism and concomitant changes in viability, but that both parameters in most cases are not tightly correlated.

## Materials and Methods

A series of cryostress experiments was conducted using bacteria, fungi, algae, plant tissues and cell lines, as well as human cell lines, as detailed in the following paragraphs. As higher multicellular organisms cannot be cryopreserved as a whole, spores, seeds, meristematic tissue or suspended cells (cell lines) are used. Accordingly, we chose a human cancer cell line (JURL-MK1) relevant for medical research, a cell culture of the major crop plant *Solanum tuberosum* cv. Desiree, and shoot tips of the species *Arabidopsis thaliana*, which is the bona fide model organism used in plant research. We focused on the effect of established and optimized cryopreservation conditions that are currently in use for the different cells types (Supplementary Figure S1).

### Bacterial Strains

Two different bacterial genera, the Gram-positive *Planococcus* (*Pla*.) and Gram-negative *Psychrobacter* (*Psy*.) were selected for the current study. Of the genus *Planococcus*, the mesophilic species *Pla. plakortidis* DSM 23997^T^ (Kaur et al., 2012), and the psychrophilic *Pla. donghaensis* DSM 22276^T^ (Choi et al., 2007) and *Pla. halocryophilus* DSM 24743^T^ (Mykytczuk et al., 2011) were analyzed. Similarly, the mesophilic *Psy. marincola* DSM 14160^T^ (Romanenko et al., 2002) was compared to the two psychrophilic species *Psy. aquaticus* DSM 15339^T^ (Shivaji et al., 2005) and *Psy. cryohalolentis* DSM 17306^T^ (Bakermans et al., 2006). Detailed growth experiments demonstrated that all psychrophilic species could grow at subzero temperatures in contrast to their mesophilic relatives (data not shown).

*Planococcus* strains were grown in Tryptic Soy Broth (Merck) supplemented with 0.3% yeast extract (w/v, TSY), *Psy. aquaticus* in Lysogeny Broth (LB; (Bertani, 1951) and the other two *Psychrobacter* strains in Marine Broth (MB, Merck). The mesophilic strains were routinely grown at 28°C and the psychophilic strains at 20°C. Cells were harvested at the end of the exponential growth phase. Cryostress experiments were conducted in three biological replicates in a final volume of 200 μl each using 500 μl 96-deep well plates, adding 10% dimethylsulfoxide (v/v, DMSO) as a cryoprotectant to the above described media. The 96 well plates were directly frozen in the gas phase of a liquid nitrogen tank and thawed after 24 h in a 30°C water bath. ATP content, OD_600_ and colony forming units (CFUs) were determined before freezing (BF), after adding the cryoprotectant (BF_treat), directly after thawing (AF) and after regrowth under optimum conditions at the end of the exponential growth phase (RG) (Supplementary Figure S1). Total cell numbers (TCN) were calculated from OD_600_ values based on calibration factors determined for each strain. CFUs were determined by plating 25 μl of a 10^-6^-fold diluted culture suspension on the appropriate growth medium solidified with agar. Culturability values were calculated by dividing CFUs by TCN.

### Algal Strains

Five strains of green microalgae were selected based on their different sensitivity to ultralow temperatures. The genera *Chlorella* and *Chlamydomonas* occur ubiquitously, serve as model systems in algae research and are of biotechnological and industrial relevance. The cryosensitive *Chlamydomonas reinhardtii* (SAG 11-32b) and *Chlorella variabilis* (strains ATCC 30562 and NC64A) were compared to the cryotolerant *Chlorella vulgaris* (SAG 211-11b) and *Micractinium conductrix* (SAG 241.80).

*Chlorella* and *Micractinium* strains were cultivated in basal medium with beef extract (“Erddekokt+Salze+Fleisch,” ESFl, medium 1a; Schlösser, 1994) and the *Chlamydomonas* strain on Tris-Acetate-Phosphate (TAP) medium (Gorman and Levine, 1965). Axenic growth was tested in ESFl, basal medium with peptone (ESP, medium 1b; Schlösser, 1994) and in modified Bold‘ìs Basal Medium with 1.5% w/v glucose and 2% w/v proteose peptone (TOM; Nichols and Bold, 1965). All strains were grown at a temperature of 20°C using a 12 h/12 h dark/light regime of white fluorescent light (50 μE m^-2^ s^-1^). After 2 weeks of growth, cultures in the exponential growth phase were harvested for cryostress assays. *Chlorella* and *Micractinium* strains were treated with 5% DSMO (v/v) according to the protocol introduced for *Chlorella* vulgaris using a controlled rate freezer (Day et al., 2007). For *Chlamydomonas* a protocol employing 3% (v/v) methanol as cryoprotectant was used (Crutchfield et al., 1999) since DMSO destroys the delicate cell envelope of *Chlamydomonas*. All cryopreserved strains were stored in the vapor phase of liquid nitrogen for 24 h and subsequently thawed as described previously (Day et al., 2007).

ATP content was measured at the start of the cryostress experiment (BF), after addition of cryoprotectants (BF_treat), in cultures after 24 h of cryostress [washed in culture medium and kept 24 h in the dark and 24 h under standard conditions (AF)] and after thawed cultures had been incubated for 2 weeks under standard growth conditions, transferred into fresh culture medium, and grown for two additional weeks (RG). The viability of the algae cells was determined at each sampling time through live/dead staining with fluorescein diacetate (FDA).

### The Filamentous Fungus *Aspergillus nidulans*

The ubiquitous filamentous fungus *Aspergillus nidulans* is saprophytic and exhibits cold-, heat- and osmo-tolerance. It represents an established model organism in eukaryotic cell biology and was therefore chosen for the present investigation. Cultures were produced in 100 ml *Aspergillus* minimal medium (AMM; Barratt et al., 1965), inoculated with 10^6^ *Aspergillus nidulans* spores per ml and incubated for 12 h to allow for the germination of spores and formation of sufficient biomass. The resulting mycelia were frozen at -80°C without cryoprotectant at a rate of 1°C min^-1^ using Mr. Frosty^TM^ (Nalgene^®^) and samples were then stored frozen for 4 h. Since physiological activity of microorganisms has been found to cease ≤-70°C (Christner, 2002), the results obtained could be compared to those of the other organisms. Afterwards, cells were thawed in a water bath at 37°C for 150 s (i.e., at a rate of 46.8°C min^-1^). For recultivation, mycelia were transferred into fresh AMM. For determination of ATP concentrations, mycelia were harvested at four different time points: after initial incubation under optimal growth condition (37°C for 12 h) (BF), after freezing (BF_treat), after thawing (AF) and after the recultivation of the cells for 12 h in AMM (RG). Oxygen consumption was monitored as an indicator of cell viability since viability measurements for cells in complex mycelia via colony forming units is not feasible. Mycelia were harvested at the beginning (BF) and after the cryostress and cell recovery (RG). The mycelium was transferred to fresh medium, overlaid with paraffin oil to prevent oxygen diffusion from air, and incubated at 37°C with gentle shaking (80 rpm). Oxygen concentrations were measured in time intervals of 15 min with an oxygen sensor (Oxygen Sensor Spot, SP-PSt3-NAU, PreSens Precision Sensing, Regensburg, Germany). Afterwards, dry cell masses were determined after separation of mycelia from the supernatant by filtering with Miracloth (Merck Millipore) and 5 days of drying at 60°C.

### *Arabidopsis thaliana* Tissues

For cryostress experiments of *Arabidopsis thaliana*, the established cryopreservation protocol was employed (Stock et al., 2017). Briefly, excised shoot tips were immersed overnight in liquid Murashige and Skoog medium (MS; Murashige and Skoog, 1962) containing 0.1 M sucrose, and the tips subsequently dehydrated for 20 min in MS medium containing 2 M glycerol and 0.4 M sucrose. This solution was then replaced by plant vitrification solution (PVS2; consisting of 30% w/v glycerol, 15% w/v ethylene glycol, 15% w/v dimethyl sulfoxide, 0.4 M sucrose in MS, pH 5.8) for 1 h at 4°C in the dark. After droplet vitrification by LN_2_ shock freezing, shoot tips were heated to 22°C and placed onto recovery medium [0.1 M sucrose, 0.5 mg L^-1^ zeatin riboside, 0.2 mg L^-1^ gibberellic acid (GA_3_), 0.5 mg L^-1^ indole-3-acetic acid, 1% agar in MS medium, pH 5.8]. For regeneration, the explants were maintained for 3 days in the dark at 22°C, then for 4 days under low light, long day conditions (16 h-photoperiod, irradiance 20–30 μmol m^-2^ s^-1^ at 22°C, 8 h in the dark at 20°C), and finally for additional 18 days under normal light conditions (16 h-photoperiod, irradiance 150 μmol m^-2^ s^-1^ at 22°C, 8 h in the dark at 20°C). During regeneration, the cells of plant shoot tips die and a new plant develops from meristem cells. After this recovery period, all explants lacking signs of development were considered as dead, while those which formed incomplete structures were classified as surviving; the third category (recovered) represented those tips which developed into normal plantlets. Only recovered plantlets were included in the statistical analyses. All values reported represent the mean of three replicates, each of which comprised a group of 30 shoot tips.

### *Solanum tuberosum* Cell Line

The wild type of *Solanum tuberosum* cv. Désiree (DSMZ No. PC-1182) was subcultured in 300 ml Erlenmeyer flasks with 100 ml cell suspension at 23°C and harvested as previously described (El-Banna et al., 2010). For cryostress experiments suspended cells were taken from the logarithmic growth phase 3 days after the last transfer. The controlled-rate cooling approach based on the method of Withers and King (Withers and King, 1980) was applied using the minitest system (Heine-Dobbernack et al., 2008) and the protocol described by Vaas et al. (2012). Cell suspensions were pretreated for 48 h with sorbitol (final concentrations, 0, 0.3, 0.6, and 1.2 M) then incubated in 5% (v/v) DMSO for 1.5 h, cooled at rate of -0.25°C min^-1^ to -40°C, and finally immersed in liquid nitrogen. After storage in liquid nitrogen for 1 day, samples were rapidly thawed at 40°C in a water bath. All individual experiments were conducted in six biological replicates.

Cellular ATP content and viability of the cells were determined after sorbitol and DMSO pretreatment (BF_treat), directly after thawing (AF), as well as after 1 week (RG1) and 5 weeks (RG5) of regeneration in 4X medium (Gamborg et al., 1968) containing 2 mg L^-1^ 2,4-dichlorophenoxyacetic acid, 0.5 mg L^-1^ indole-3-acetic acid, 0.5 mg L^-1^ 1-naphtylacetic acid and 0.4 mg L^-1^ kinetin. Untreated inoculum cells from the logarithmic growth phase were used as reference (BF). Viability was determined by Evans Blue staining modified after Suzuki et al. (1999). Plant cells (150–300 mg fresh weight) were stained for 5 min with 0.5% Evans Blue in 4X medium. Afterwards cells were washed four times with 4X medium (BF, RG1, and RG5 cells) or with 4X medium supplemented with the respective sorbitol concentration (BF treat and AF cells). For Evans Blue staining of regrown cells the content of the cryotubes was poured onto 4X agar plates covered with three sterile filter paper disks. After 2 h of incubation the uppermost filter was transferred to fresh 4X agar. All agar plates were incubated in the dark at 23°C and the percentage of viable cells was determined after 1 and 5 weeks, respectively.

### Human Cell Line JURL-MK1

The human chronic myeloid leukemia cell line JURL-MK1 was obtained from the repository of the Leibniz Institute DSMZ, and cultured in humidified air at 5% CO_2_ and 37°C in 80% RPMI 1640 medium (Life Technologies) supplemented with 20% (v/v) fetal bovine serum (FBS) (Sigma). Cell cultures were maintained at 0.5 to 2.0 × 10^6^ cells ml^-1^. For cryostress experiments, four million cells of an exponentially growing culture (sample BF) were harvested and resuspended in 1 ml of freezing medium (70% RPMI 1640, 20% FBS, 10% v/v DMSO) per cryotube (sample BF_treat). These cell suspensions were frozen at a rate of -1°C min^-1^ until -80°C using a freezing container (Mr. Frosty^TM^, Nalgene^®^) and subsequently transferred to the vapor phase of liquid nitrogen for storage. After 4–6 days, cryotubes were rapidly thawed in a water bath at 37°C and cell suspensions immediately diluted 10-fold with complete culture medium (sample AF). Thawed cells were then sedimented, the supernatant containing freezing medium discarded, and cells subcultivated at a density of 0.8 × 10^6^ cells ml^-1^ as described above (sample AF). Finally, survival and regrowth were determined 3 days post thawing (sample RG). At each sampling point, aliquots were subjected to vital staining using trypan blue (Sigma) to determine cell density and viability using a Neubauer chamber (Brand GmbH & Co Kg).

### ATP Assay

ATP was measured using the Bac- or CellTiter-Glo^®^ Luminescent Cell Viability Assay (Promega) according to the instructions of the manufacturer. Bacterial cultures were diluted 10-fold and human cell suspensions adjusted to 0.8 × 10^6^ viable cells ml^-1^ and 50 μl of the reagent were added to 50 μl of cell suspension in white opaque 96-well plates (Greiner). For algae, fungi, plant cell lines and plant tissue, specifically adapted ATP extraction protocols had to be applied prior to ATP measurement.

Algal and fungal cells (500 μl re-suspended pellet of 50 ml algae cultures corresponding to 3–5 × 10^7^ cells ml^-1^) and fungal mycelia were mechanically disrupted in liquid nitrogen. ATP was extracted from 100 to 200 mg cell mass by mixing with 400 μl Tris/EDTA-buffer (0.1 M Tris-Acetate-Buffer, 2 mM EDTA, pH 7.75) and 500 μl cold (4°C) TCA/EDTA-solution (10% w/v trichloroacetic acid, 4 mM EDTA). The emulsion was centrifuged (10 min at 4°C, 20.000 × *g*) and 20 μl of the supernatant were transferred into a 96 well plate and four 10-fold serial dilutions were prepared in Tris/EDTA-buffer. 100 μl Tris/EDTA and 100 μL BacTiter-Glo reagent buffer were added to each sample.

For plant cell lines and tissue, ATP extraction was performed as described in (Smyth and Black, 1984) with small adaptations. 150 to 300 mg (fresh weight) plant cells were supplemented with 0.3 ml cold perchloric acid (0.83 M) and homogenized for 30 s. Another 0.7 ml cold perchloric acid were added and samples centrifuged for 15 min at 4°C and 10.000 × *g*. Then 225 μl Bicine (1 M, pH 7.75) were added to 900 μl of the supernatant, the sample was immediately placed on ice and the pH adjusted to 7.6–8.0 by addition of 4 M KOH. The white precipitate was centrifuged for 10 min at 4°C. 100 μl BacTiter-Glo^®^ reactant was added to 100 μl of extract.

Luminescence of all samples was measured with an integration time of 0.5 s and automatic attenuation using a luminescence plate reader (Tecan Infinite M200). Samples were measured in technical duplicates (or triplicates for human cells) and ATP-standard curves with ATP diluted in culture medium or the respective extraction buffer included in each measurement. The amount of cellular ATP was calculated in moles per biomass. To calculate the ATP amount per mg cell material, the cell mass (dry weight), the protein concentration (Bradford, 1976) or the whole protein amount per biomass (dry weight) were determined depending on the organisms.

### Statistics

For the measured parameters (cellular ATP and viability), the means of three replicates (4–6 parallels for the plant cell line) and 95% family wise confidence levels were calculated with R version 3.4.3 (R Core Team, 2017). Significant differences between consecutive sampling time points were calculated by one-way-ANOVA with multiple comparisons of means using Tukey Contrasts (package multcomp; Hothorn et al., 2008) and shown as compact letter display (cld; Piepho, 2004). Correlations for the association between paired samples were tested (R, corr.test) using two-sided Spearman’s rank correlation rho. Distinct patterns in ATP content across the model organisms under cryostress, were evaluated employing shape-based time-series clustering (R, dtwclust; Sardá-Espinosa, 2017; Sardá-Espinosa et al., 2017) that refers to the Dynamic Time Warping distance (DTW; Keogh and Ratanamahatana, 2005; Lemire, 2009). Five different clustering algorithms [DTW basic, DTW with window of 1 and shape as centroid, cluster κ-shape, Time-series Anytime Density Peaks Clustering and a distance based on Global Alignment Kernels (GAK) with shape as centroid] using two to six clusters were evaluated using internal cluster validity indices. A particular algorithm was identified as optimum, if at least three of the indices yielded the best results.

## Results

### Cellular ATP as an Indicator for Cryostress of Different Groups of Organisms

Cellular ATP concentrations were used to probe the physiological responses of the different cell types to optimum growth conditions (BF), to cryoprotectant (BF_treat), deep freezing (AF), and after regeneration of the cells from cryostress (RG). Methods of cryopreservation that had previously been established for each group of organisms were applied (Supplementary Figure S1) in order to compare the cryostress response across all cell types under their specific, currently optimized conditions.

**FIGURE 1:**
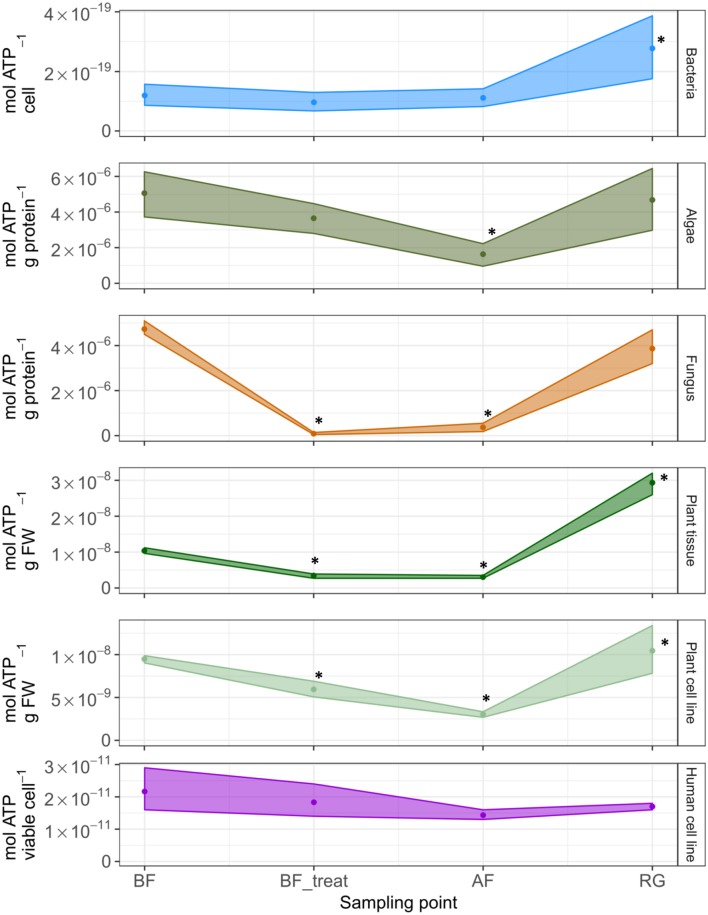
Changes in intracellular ATP levels observed for the different model organisms during cryostress experiments. Points denote the mean ATP content of all representatives of a group of organisms at the individual sampling points. 95% confidence intervals are displayed as ribbons. Values that were significantly different (*p* < 0.05) from the BF value within a group of organisms are marked with ^∗^. FW, fresh weight; BF, before freezing; BF_treat, after treatment with cryoprotectant; AF, after freezing; RG, after the regrowth phase. Compare Supplementary Figure S1 for details on individual sampling points.

For all groups of organisms, a decrease in the specific ATP content was observed during cryostress experiments. Minimum values were determined upon treatment with cryoprotectant (BF_treat) and/or after the freezing period (AF) (**Figure 1** and Supplementary Table S1). When data were aggregated groupwise, this decline in cellular ATP content was significant for all groups of organisms except for the combined six bacterial strains and for the human cell line (**Figure 1**, asterisks). After the phase of regrowth, bacterial cells and plant tissue even reached higher cellular ATP levels than the initial cultures (**Figure 1** and Supplementary Table S1). Thus, the initial ATP was exceeded up to four times in bacteria (*Psy. marincola*), up to three times in *Arabidopsis thaliana* shoot tips and 1.5 times in *Solanum tuberosum* cell lines treated with 1.2 M sorbitol (Supplementary Table S1). Based on these groupwise data, deep freezing was the single step during the cryostress experiment that affected the cellular ATP content most pronouncedly across almost all groups of organisms.

### Changes in the Cellular ATP Content and in Viability of Different Microbial Strains

**FIGURE 2:**
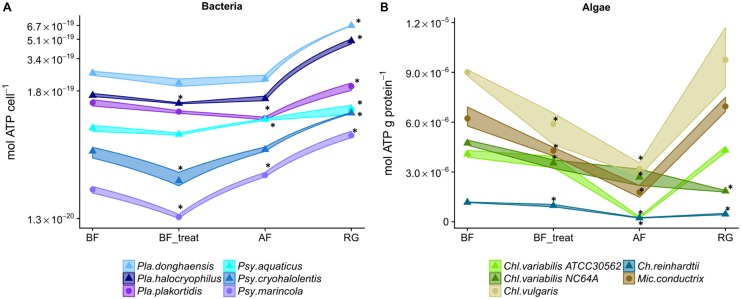
Strain-specific alterations in intracellular ATP levels during cryostress experiments of bacteria and green algae. ATP content of cells of individual strains before freezing (BF), after the addition of a cryoprotectant (BF_treat), after freezing and thawing (AF) and after a regrowth phase (RG). Depicted are the means of three biological replicates with 95% confidence intervals presented as ribbons. **(A)** Response of the six different bacterial strains. **(B)** Response of the five different strains of green algae. 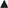 psychrotolerant bacteria/cryotolerant algae, ● mesophilic bacteria/cryosensitive algae. ^∗^ denotes values that were significantly different (*p* < 0.05) from the BF value of the respective strain. *Pla.*, *Planococcus*; *Psy.*, *Psychrobacter*; *Ch.*, *Chlamydomonas*; *Chl.*, *Chlorella*; *Mic., Micractinium.*

During the cryostress experiment all six bacterial strains studied exhibited similar patterns of changes in their cellular ATP content (**Figure 2A** and Supplementary Table S1). This is also corroborated by 12 out of the 15 possible pairwise correlations of ATP contents that proved to be highly significant (Supplementary Table S2). The addition of DMSO before freezing (step BF_treat) led to a significant decrease of ATP levels in *Pla. halocryophilus, Psy. cryohalolentis*, and *Psy. marincola*. Directly after thawing (step AF), ATP levels were significantly elevated in *Pla. plakortidis*, *Psy. marincola*, and *Psy. aquaticus* (**Figure 2A** and Supplementary Table S1).

**FIGURE 3:**
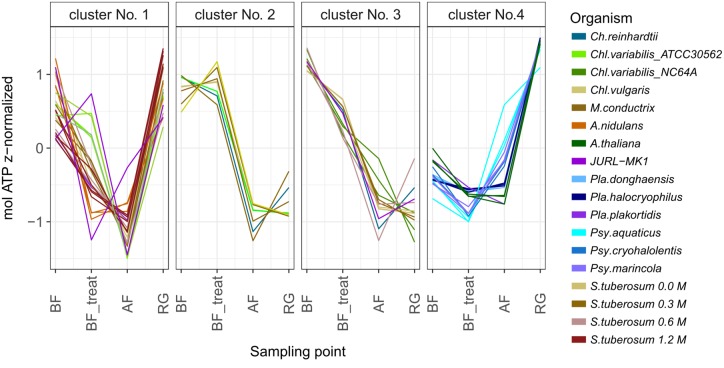
Patterns of changes in intracellular ATP content observed for different organisms during cryostress. The pattern of ATP content observed under cryrostress for each strain or organism was fitted to different shapes employing the R package dtwclust. This analysis resulted in clusters of strains or organisms that could be assigned to four different shapes (Supplementary Figure S2). BF, before freezing; BF_treat, after treatment with cryoprotectant; AF, after freezing; RG, after the regrowth phase. *Pla.*, *Planococcus*; *Psy.*, *Psychrobacter*; *Ch.*, *Chlamydomonas*; *Chl.*, *Chlorella*; *Mic., Micractinium; A. nidulans, Aspergillus nidulans; A. thaliana, Arabidopsis thaliana* Col-0 shoot tips; *S. tuberosum, Solanum tuberosum* cv. Desiree suspension cells after pretreatment with different sorbitol concentrations.

In order to verify and classify the distinct patterns in ATP content across the different model organisms under cryostress, the pattern determined for each strains or organism was fitted to different shapes employing the R package dtwclust. The GAK algorithm performed best to describe the observed trends in cellular ATP. This analysis resulted in clusters of strains or organisms that could be assigned to four different shapes (Supplementary Figure S2). Most bacterial strains fell into a cluster characterized by a pattern of limited decrease in ATP concentrations upon DMSO addition and a pronounced increase in ATP content after regeneration (cluster 4; **Figure 3** and Supplementary Table S1). Only *Pla. plakortidis* did not exhibit this pronounced final increase in ATP and hence fell into cluster one (**Figure 3**).

**FIGURE 4:**
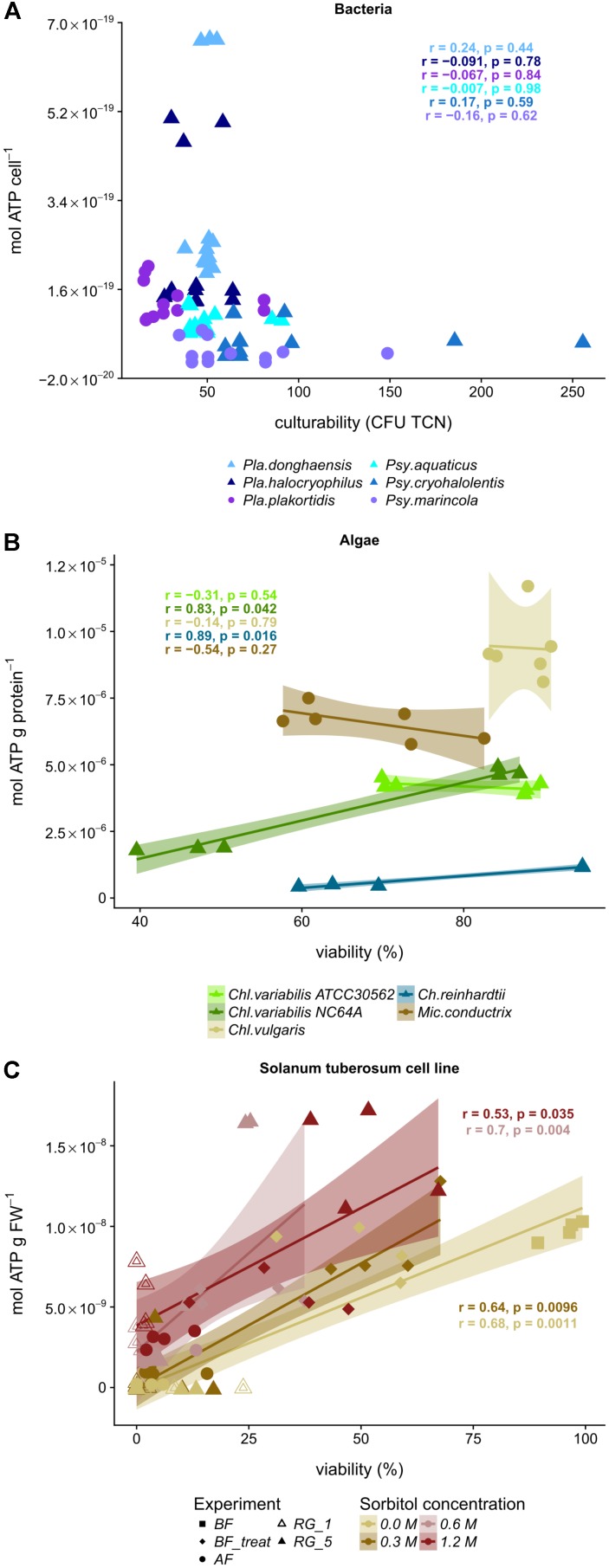
Correlation of ATP levels and viability during freezing of bacteria, green algae and a plant cell line. **(A)** Depicted is the ATP content vs. culturability (CFU, colony forming units per TCN, total cell numbers) of the different bacterial strains (*Pla*., *Planococcus*, *Psy*., *Psychrobacter*). **(B)** ATP content vs. viability with 95% confidence interval and linear regression of the green algae *Ch*., *Chlamydomonas*; *Chl*., *Chlorella*, and *Mic*., *Micractinium.* 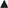 psychrotolerant bacteria/cryotolerant algae, ● mesophilic bacteria/cryosensitive algae. **(C)** ATP content vs. viability with 95% confidence interval and linear regression of suspension cultures of *Solanum tuberosum* cv. Desiree after pretreatment with different sorbitol concentrations before freezing (BF), after the addition of a cryoprotectant (BF_treat), after freezing and thawing (AF) and after a regrowth phase (RG). r, Spearman’s rank correlation, estimated measure of the association; p, *p*-value.

Notably, culturability and ATP contents were not correlated in cryostress experiments with bacterial strains (Supplementary Table S2 and **Figure 4A**). After regeneration, the initial culturability was reached in the psychrotolerant species *Pla. donghaensis* and *Pla. halocryophilus*, but this did not mirror the distinctively elevated ATP contents. For the mesophilic *Pla. plakortidis*, both, culturability and ATP content, declined after freezing and for the mesophilic *Psy. marincola*, both, culturability and ATP content, were elevated after freezing, but the ATP content was not commensurate with culturability after regeneration. While the ATP content was increased after regeneration, culturability dropped below the initial point. Overall, the culturability of mesophilic bacteria in this study was found to be lower after the final regeneration than for psychrophilic.

The cellular ATP concentrations of green algae followed two principles and distinct patterns during cryostress experiments. One cluster of strains (No. 1, **Figure 3**) exhibited a strong ATP decline after freezing and thawing, followed by an increase in ATP content after regrowth which recovered fully back to initial levels. This cluster comprised the cryotolerant *Chl. vulgaris*, *Chl. variabilis* A, and *Mic. conductrix* (**Figures 2B**, **3**). Interestingly, cellular ATP content of the cryosensitive strains *Ch. reinhardtii* SAG 11-32b and *Chl. variabilis* NC64A did not return to the high initial values even after regrowth but instead reached only 40% of these levels (**Figure 2B**); these strains thus fell into another cluster of strains with a different pattern (no. 3, **Figure 3**). Thus, the two *Chl. variabilis* strains showed similar ATP contents before freezing (4.08 × 10^-06^ and 4.74 × 10^-06^ mol ATP g protein^-1^ in ATCC30562 and NC64A, respectively) and also after cryoprotectant treatment, but differed in later stages of the cryostress experiments. The extent of ATP decrease during freezing and thawing was more pronounced in the cryotolerant ATCC strain but cellular ATP levels were completely restored after regrowth, whereas the ATP levels of NC64A remained below the values of starting cultures. Cell size and ATP content per g protein were negatively correlated within the different algae species (*p* = 0.038, *R*^2^ = 0.742, estimate = -0.9, linear regression calculated with Pearson’s product-moment correlation), due to the relatively higher protein amount of the motile *Chlamydomonas* algae (Ø 4.6 μg ml^-1^), compared to *Chl. vulgaris* (Ø 1.4 μg ml^-1^) or *Chl. variabilis* (Ø 3.6 μg ml^-1^). Therefore viability and ATP content had to be compared for each strain separately. The ATP content was significantly correlated to viability in the cryosensitive *Ch. reinhardtii* and *Chl. variabilis* NC64A (**Figure 4B** and Supplementary Table S1), whereas viability does not reflect the ATP content of the cells in *Chl. variabilis* ATCC 30562, *Chl. vulgaris* and *Mic. conductrix*.

The cellular ATP content of the fungus *Aspergillus nidulans* exhibited a pattern similar to that of cryotolerant green algae (cluster with pattern No. 1; **Figure 3** and Supplementary Table S1). After a pronounced decline of the cellular ATP content during freezing and thawing, ATP levels were fully restored after the regeneration period of 12 h and no significant differences to the values of the first sampling time point were observed (**Figure 2**). At optimum growth, the oxygen consumption rate per dry weight was significantly lower (*p* < 0.01) in comparison to the cells regenerated after thawing. Since the decrease of the oxygen consumption (i.e., the respiration rate) stayed constant over the whole course of the cryostress experiment, it has to be concluded that no significant cell death occurred. The oxygen consumption rate did not correlate with the ATP content (Supplementary Table S2).

Taken together, the results obtained for the 12 different types of microorganisms clearly demonstrate that the cellular ATP contents do not mirror the cell viability (Supplementary Table S2). In most cases, bacteria and algae showed high ATP levels after regrowth regardless of a lower viability. A significant positive correlation of ATP content and viability was determined only under optimal conditions (BF) and for the cryosensitive algae *Chlamydomonas reinhardtii* and *Chl. variabilis* NC64A as well across experiments.

### Cryostress Response of Cells From Multicellular Eukaryotes

**FIGURE 5:**
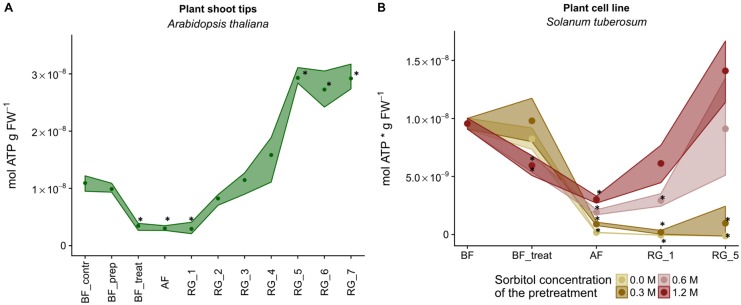
Strain-specific responses in intracellular ATP concentrations during cryostress experiments of plant shoot tips and a plant cell line. ATP content of cells from the individual strains before freezing (BF), after the addition of a cryoprotectant (BF_treat), after freezing and thawing (AF) and after a regrowth phase (RG). Depicted are the means of the biological replicates with 95% confidence intervals represented as ribbons. **(A)** Response of *Arabidopsis thaliana* Col-0 shoot tips. BF_contr, control (wild-type seedlings); BF_prep, prepared (freshly excised shoot tips); RG_1 – RG_7, regrowth after 1–7 days. **(B)** Response of *Solanum tuberosum* cv. Desiree suspension cells after pretreatment with different sorbitol concentrations. RG_1 and RG_5, regrowth after 1 and 5 week(s). ^∗^ denotes values that were significantly different (*p* < 0.05) from the BF value of the respective strain. FW, fresh weight.

The ATP concentration of *Arabidopsis thaliana* in wild-type seedlings (1.09 × 10^-8^ mol ATP g FW^-1^) and freshly excised shoot tips (9.89 × 10^-8^ mol ATP g FW^-1^) did not vary significantly (Supplementary Table S2). The ATP level during cryopreservation was characterized by an ATP decline after cryoprotectant treatment and after freezing and thawing and this was similar to the patterns observed for bacteria or the fungus (**Figures 3**, **5A**). Within the first 3 days of regeneration (RG_3), the ATP level increased steadily to concentrations comparable to that of the seedling stage (1.15 × 10^-8^mol ATP g FW^-1^) (compare Supplementary Figure S3A) but continued to increase until day 5 when even higher levels were attained (**Figure 5A**) accompanied by a rapid development of the seedlings (Supplementary Figure S3B). The viability after a regeneration of 25 days was close to 100% (Supplementary Table S1).

Plant cell lines are especially vulnerable to cryostress due to their high water content and the presence of water in large vacuoles. Since cryopreservation of plant cell lines involves specific pretreatment with sorbitol, the effect of the resulting osmotic stress was investigated systematically, using cell suspensions of *Solanum tuberosum* cv. Désiree. Before freezing and under optimal growth conditions, the ATP content was 9.6 nmol ATP (g FW)^-1^ and 96% of the cells were viable (Supplementary Table S1). Already in the absence of sorbitol, addition of DMSO decreased the viability significantly to 50% (Supplementary Figure S3C). Pretreatment of the cells with 0.6 or 1.2 M sorbitol reduced the ATP content significantly to about 60% (**Figure 5B**), after pretreatment of the cells without or 0.3 M sorbitol the decrease was not significant based on multiple comparisons by ANOVA-testing. The lower viability of cells pretreated with these higher sorbitol concentrations was not significant as compared to cells exposed to no or lower concentrations of sorbitol (Supplementary Table S1, cld “c”). Interestingly, the decreases in cellular ATP content during deep freezing and after regeneration were inversely correlated with the concentrations of sorbitol during pretreatment (**Figure 5B** and Supplementary Table S1). Thus, the ATP contents of cells without sorbitol or pretreated with 0.3 M sorbitol did not recover even in later stages of regeneration (i.e., clustered with patterns 2 and 3, **Figure 3**) whereas the higher sorbitol concentrations resulted in elevated cellular ATP content at the end of the cryostress experiment (pattern 1, **Figure 3**). Viability values showed a similar massive decline and dropped to ≤6% during deep freezing but unlike the ATP content remained low also during the subsequent 1st week of regeneration (Supplementary Table S1). After 5 weeks of regeneration, viability values had recovered to 51% only in cultures pretreated with 1.2 M sorbitol, reached lower values (mean, 16%) after pretreatment with 0.6 M sorbitol but remained at low levels for the other cultures. These significant differences indicate a major effect of sorbitol pretreatment on the survival of plant cell during cryostress (Supplementary Table S1). Due to the different kinetics of ATP concentrations and viability of the cells, both parameters were not significantly correlated for cells treated with 0.6 and 1.2 M sorbitol (**Figure 4C**).

In the cancer cell line JURL-MK1, cellular ATP levels remained stable during freezing and thawing and the observed differences were not statistically significant (**Figure 1** and Supplementary Table S1). Also, the freezing and thawing steps had no considerable effect on cell viability which remained constantly high >95% (Supplementary Table S1).

## Discussion

So far, the physiological responses to temperature decrease have mostly been studied under cold stress rather than for ultradeep freezing conditions. Few individual organisms have been tested at subzero temperatures (Christner, 2002; Junge et al., 2006; Morrison and Shain, 2008; Amato and Christner, 2009), but in these cases changes in ATP concentrations and viability have not been determined together. In particular, the effects of cryopreservation on the physiological state of the cells have not been assessed systematically. Our comparative cryostress experiments employed established cryopreservation procedures and monitored the resulting changes in cellular ATP and their relation to cell viability across a wide range of different types of organisms.

The intracellular ATP concentrations determined for growing cells in the present study are comparable to those in the literature [10^-19^ mol ATP cell^-1^ for *Psy. cryohalolentis* (Amato and Christner, 2009); 37.6–161.4 μmol ATP mg chlorophyll a^-1^ h^-1^ for algae (Sawa et al., 1982), 1–17 μmol ATP mg chlorophyll a^-1^ in our study; 6 × 10^-6^ mol ATP g dry weight^-1^ for *Saccharomyces cerevisiae* (Thomsson et al., 2003); 30–80 nmol g FW^-1^ for maize cell cultures (Sowa et al., 1998); 50 nmol g FW^-1^ in *Arabidopsis* seedlings (Zhu et al., 2012); 3.95 × 10^-11^ mol ATP cell^-1^ for a chronic myelogenous leukemia cell line (Mikirova, 2015)]. Considerable differences in the cellular ATP contents were observed between the six bacterial strains investigated in this study. Since the cellular ATP concentrations were correlated significantly (*p* = 0.044, *R*^2^ = 0.596, estimate = 0.82, linear regression calculated with Pearson’s product-moment correlation) with the respective cell volumes of the strains (0.39–1.02 μm^3^ × cell^-1^), the differences in cellular ATP content can be explained by the different size of the bacteria. Cell size and ATP content (determined per g protein) were negatively correlated for the different algae species, which most likely is due to the motility and the higher relative protein content of the larger *Chlamydomonas* algae.

Cold shock (i.e., a decrease of incubation temperature by 10–20°C below temperature for growth optimum, for 120 min) has been demonstrated to result in a rapid loss of ATP and total adenylate nucleotides in mesophilic bacteria, fungi and protists, but on the opposite leads to increased intracellular concentrations in psychrophilic representatives, including *Psy. cryohalolentis* which was investigated also in the present study (Napolitano and Shain, 2005; Amato and Christner, 2009). It has therefore been suggested that the mode of regulation of the adenylate pool differs in psychrophiles, and enables maintenance of elevated ATP concentrations that offset reductions in molecular motion and Gibb’s free energy of ATP hydrolysis (Morrison and Shain, 2008). In plant cells, the physiological adaptations during cold acclimation (so-called “hardening”) require several weeks of incubation at non-freezing temperatures (Sobczyk and Kacperska-Palacz, 1978; Uemura and Steponkus, 1994). Winter rape (*Brassica napus* L. var. *oleifera* L.) plants maintain intracellular ATP concentrations in the dark upon cooling to 0°C. After cold acclimation, freezing results in increased ATP concentrations in leaves but decreased concentrations in roots (Sobczyk and Kacperska-Palacz, 1978).

In contrast to these previously published results, increases of cellular ATP concentrations at ultradeep temperatures were never observed for any of the cell types tested. Instead, representatives of bacteria, fungi, algae, plant tissue, and plant cell lines directly subjected to ultradeep freezing all showed significant declines in cellular ATP levels which in most cases recovered to the initial or even higher values upon regeneration. In bacteria and plant (tissue) ATP levels during regeneration exceeded those before freezing by upto four times. This increase can be attributed to a higher energy demand during exponential growth phase and the development of new tissue (Morita and Morita, 1997). The plant shoot tip cells die during regeneration and a new plant develops from the meristem cells. This cell differentiation and active metabolism are typically characterized by elevated ATP levels (Rolletschek et al., 2004).

Most biochemical processes cease when ambient temperatures fall below -70 to -80°C (Christner, 2002; Junge et al., 2006). At -40°C, bacterial metabolism is very low and appears to be limited to the repair of macromolecular damage of the largely dormant cells (Price and Sowers, 2004). Previously, increases in cellular ATP concentrations of psychrophilic bacteria upon exposure to subzero temperatures were observed when employing slow cooling rates [0.32–1.91°C min^-1^; (Amato and Christner, 2009)] that may elicit a physiological response of the bacterial cells while still in liquid cultures. Similarly, the time course of cooling used in established cryopreservation protocols (Supplementary Figure S1) is much too short to allow for cold-adaptation of plant cells and tissues. Reduced ATP values as a consequence of cryoprotectant exposure have been reported in catfish spermatozoa (De Baulny et al., 1999), gorgonian coral (Tsai et al., 2010, 2014a,b, Lin et al., 2011) and porcine oocytes (Tsuzuki et al., 2009), but did not seem to occur in the human cell line when the established cryopreservation procedures were employed. Furthermore, cryoprotectant treatment was avoided in starved psychrophilic bacteria after incubation at 22°C for 88 h before cryostress (Amato and Christner, 2009). In addition, cryoprotectants and plant vitrification solutions have been reported to induce hypoxic stress in plant tissues, such as garlic shoot tips (Subbarayan et al., 2015). Similarly, an ATP decrease has been related to hypoxia during barley seed development (Rolletschek et al., 2004). Mitochondrial dysfunction resulting in hypoxia and apoptosis of retinal ganglion cells could be prevented by taurine treatment of the cells, accompanied by cellular ATP loss (Chen et al., 2009). Furthermore, mitochondria of maize seedlings have shown to be a target for chilling-induced oxidative stress that finally impairs cellular respiration and ATP formation (Prasad et al., 1994). Thus, cryopreservation related stressors, such as oxidative stress or cryoprotectants, can cause mitochondrial dysfunction leading to hypoxia, accompanied by ATP depletion.

Different biochemical processes are known to affect cellular ATP content upon decreasing temperatures. In bacteria, futile cycling of protons or other ions (K^+^, NH_4_^+^) occurs if ATP-consuming active transport is neutralized by reverse transmembrane movements due to turgor-activated membrane channels or changes in membrane resistance, thereby causing a pronounced decrease in cellular ATP on a short-term scale (Russell and Cook, 1995). In leaves of winter rape, the decrease in intracellular ATP concentrations is associated with changes in membrane permeability, initially through the inactivation of ion-dependent ATPases. Subsequent irreversible membrane damages result in degradation of ATP (Sobczyk et al., 1985). No significant changes in cellular ATP content could be observed for the continuous human JURL-MK1 cell line. In contrast, primary human peripheral blood leukocytes have been shown to be compromised in mitochondrial function and ATP production during cryopreservation (Keane et al., 2015). Hence, in eukaryotic cells, mitochondrial function may be affected by cryostress, while the plasma membrane remains intact. Slow cooling of human umbilical vein endothelial cells resulted in depolarization and dysfunction of mitochondria immediately after thawing (Reardon et al., 2015), whereby the detailed mechanisms of the indirect effects of cooling on cell organelles need further investigations. However, the observation of a consistent decline in intracellular ATP concentrations observed across almost all other organisms, independent of their psychrophilic, psychrotolerant, or mesophilic character, together with the speed of temperature decline, indicate that compensatory physiological responses to cold stress did not occur at the rapid freezing rates used in the present study. Rather, constitutive biochemical processes dominated the energetic state of the cells during cryopreservation as well as the impairment of cellular respiration.

The goal of the established, often complex cryopreservation techniques is to maintain as many cells as possible in a viable state that ensures growth upon thawing. In order to investigate whether ATP is a major determinant of cell survival (Pegg, 1989), we systematically tested the correlation between cellular ATP content and viability during different stages of cryopreservation and across different types of organisms. Such a correlation is suggested by some previously published observations. Elevated ATP concentrations induced in an *Escherichia coli* knockout mutant resulted in a significantly increased survival after exposure to 0°C compared to the wild type (Morrison and Shain, 2008). Other stresses [such as anaerobic carbon starvation in the yeast *Saccharomyces cerevisiae*; (Thomsson et al., 2003) which result in energy deprivation and drastically reduced intracellular ATP-concentrations] also cause a major loss in physiological activity and viability upon subsequent substrate addition. Yet, the correlation between ATP content and biomass formation in bacteria is often poor due to maintenance energy requirements and other ways of non-growth energy dissipation, the latter can exceed the former by an order of magnitude (Russell and Cook, 1995).

The lack of correlation between cellular ATP content and viability after ultradeep freezing was observed across almost all types of organisms covered in the present study. A link between cellular ATP content and viability was only observed for two cryosensitive alga and plant cell cultures kept at low osmotic pressures, in which ATP concentrations did not return to pre-cryostress levels, suggesting irreversible damage of the cells under the conditions applied. Our results support the conclusion that factors other than ATP content determine changes in cell viability as long as cryopreservation does not irreversibly affect the cells. This is also commensurate with the observation that an increased respiration rate occurred during the regeneration of *Aspergillus nidulans*, enabling the return to initial values of cellular ATP values. Based on our results, adaptations to cold stress do not occur during cryopreservation. Instead, the physiological response and survival of almost all cell types is determined by other, constitutive processes such as changes in membrane permeability and ion transport. The cold stress response may provide additional opportunities for improving cryopreservation techniques but to our knowledge, this has not been studied systematically to date. From a practical point of view, our results clearly indicate that intracellular ATP content cannot be used as a reliable predictor of cell viability for further optimization of cryopreservation for the organisms tested in the present study.

## Author Contributions

JO, OK, H-PM, SE, TF, HS, EK, ML, and FB designed the study. FB, JS, BH, TD, EH-D, and LN performed parts of the lab work and mainly analyzed and interpreted the data. JO, OK, H-PM, SE, TF, HS, EK, and ML substantial contributed to the interpretation of the results and valuable discussion. FB was drafting the work. JS, BH, TD, EH-D, and SE contributed the drafting. JO, OK, H-PM, TF, HS, EK, and ML revised it critically for important intellectual content. All authors finally approved the version to be published and agreed to be accountable for all aspects of the work in ensuring that questions related to the accuracy or integrity of any part of the work are appropriately investigated and resolved.

## Conflict of Interest Statement

The authors declare that the research was conducted in the absence of any commercial or financial relationships that could be construed as a potential conflict of interest.
