# Supplementary material for: ATP Content and Cell Viability as Indicators for Cryostress Across the Diversity of Life
Source: Front Physiol. 2018 Jul 17;9:921. doi: 10.3389/fphys.2018.00921 (PMC6056685; doi:10.3389/fphys.2018.00921)
Supplement: Supplementary file 1 [file Table_1.DOCX]

Table S1. Cellular ATP content and viability of bacteria, green algae, a fungus, a plant cell line, plant tissue, and a human cell line. For each organism, the significance of differences of the measured parameters (cellular ATP and viability) between consecutive sampling time points during the cryostress experiments were calculated (by ANOVA) and shown as compact letter display (cld). The responses of ATP content cluster in 4 different patterns (clusters 1-4, compare Fig S2). CI 95 % confidence interval, CFU colony forming unit, TCN total cell number, nd not determined, BF before freezing, BF_control, BF_treat after treatment with cryoprotectant, AF after freezing and thawing, RG after a phase of regrowth, * significant difference in comparison to BF, p<0.05, FDA fluorescein diacetate, FW fresh weight, DW dry weight.

| **Organism**  Specific strain/cell line | **Experiment**  **_timepoint** | **Replicates** | **mol ATP** | **CI mol ATP** | **cld**  **ATP** | **cluster** | **Viability**  **(%)** | **CI viability** | **cld**  **viability** |  |
| --- | --- | --- | --- | --- | --- | --- | --- | --- | --- | --- |
| **Bacteria** |  |  | mol ATP cell^-1^ |  |  |  | Culturability (CFU TCN^-1^) | | |  |
| *Planococcus donghaensis*  DSM 22276^T^ | BF_0 | 3 | 2.56×10^-19^ | 2.66×10^-20^ | n | 4 | 51 | 4 | d |  |
|  | BF_treat_0 | 3 | 2.09×10^-19^ | 4.07×10^-20^ | mn |  | nd | nd |  |  |
|  | AF_1 | 3 | 2.27×10^-19^ | 3.96×10^-20^ | mn |  | 45 | 15 | bd |  |
|  | RG_1 | 3 | 6.70×10^-19*^ | 4.97×10^-21^ | p |  | 51 | 11 | cd |  |
| *Planococcus halocryophilus*  DSM 24743^T^ | BF_0 | 3 | 1.63×10^-19^ | 1.37×10^-20^ | kl | 4 | 50 | 29 | bcd |  |
|  | BF_treat_0 | 3 | 1.38×10^-19*^ | 3.79×10^-21^ | j |  | nd | nd |  |  |
|  | AF_1 | 3 | 1.52×10^-19^ | 1.79×10^-20^ | jk |  | 29 | 5 | b |  |
|  | RG_1 | 3 | 4.91×10^-19*^ | 6.39×10^-20^ | o |  | 42 | 37 | abcd |  |
| *Planococcus plakortidis*  DSM 23997^T^ | BF_0 | 3 | 1.40×10^-19^ | 2.24×10^-20^ | ijk | 1 | 47 | 74 | abcd |  |
|  | BF_treat_0 | 3 | 1.17×10^-19^ | 8.61×10^-21^ | hi |  | nd | nd |  |  |
|  | AF_1 | 3 | 1.02×10^-19*^ | 8.37×10^-21^ | g |  | 18 | 5 | a |  |
|  | RG_1 | 3 | 1.96×10^-19*^ | 3.64×10^-20^ | lm |  | 16 | 3 | a |  |
| *Psychrobacter aquaticus*  DSM 15339^T^ | BF_0 | 3 | 8.39×10^-20^ | 1.26×10^-20^ | f | 4 | 45 | 12 | d |  |
|  | BF_treat_0 | 3 | 7.38×10^-20^ | 4.17×10^-21^ | f |  | nd | nd |  |  |
|  | AF_1 | 3 | 1.00×10^-19*^ | 4.30×10^-21^ | g |  | 75 | 57 | abcd |  |
|  | RG_1 | 3 | 1.24×10^-19*^ | 3.05×10^-20^ | ghj |  | 45 | 20 | bcd |  |
| *Psychrobacter cryohalolentis*  DSM 17306^T^ | BF_0 | 3 | 5.24×10^-20^ | 1.47×10^-20^ | de | 4 | 65 | 12 | c |  |
|  | BF_treat_0 | 3 | 2.88×10^-20*^ | 1.17×10^-20^ | bc |  | nd | nd |  |  |
|  | AF_1 | 3 | 5.42×10^-20^ | 6.15×10^-21^ | d |  | 179 | 198 | abcd |  |
|  | RG_1 | 3 | 1.14×10^-19*^ | 2.87×10^-21^ | h |  | 74 | 40 | cd |  |
| *Psychrobacter marincola*  DSM 14160^T^ | BF_0 | 3 | 2.41×10^-20^ | 4.00×10^-21^ | b | 4 | 58 | 53 | abcd |  |
|  | BF_treat_0 | 3 | 1.38×10^-20*^ | 1.58×10^-21^ | a |  | nd | nd |  |  |
|  | AF_1 | 3 | 3.23×10^-20*^ | 3.99×10^-21^ | c |  | 101 | 109 | abcd |  |
|  | RG_1 | 3 | 7.22×10^-20*^ | 1.39×10^-20^ | ef |  | 44 | 20 | bcd |  |
| **Algae** |  |  | mol ATP g protein^-1^ |  |  |  | FDA staining | |  |  |
| *Chlamydomonas reinhardtii*  SAG 11-32b | BF_0 | 3 | 1.17×10^-06^ | 4.30×10^-08^ | d | 3 | 95 | 0 | e |  |
|  | BF_treat_0 | 3 | 9.76×10^-07*^ | 1.80×10^-07^ | c |  | nd | nd |  |  |
|  | AF_1 | 3 | 2.34×10^-07*^ | 3.90×10^-08^ | a |  | nd | nd |  |  |
|  | RG_1 | 3 | 4.66×10^-07*^ | 1.06×10^-07^ | b |  | 64^*^ | 12 | bc |  |
| *Chlorella variabilis A*  ATCC 30562 | BF_0 | 3 | 4.08×10^-06^ | 5.12×10^-07^ | gi | 1 | 88 | 3 | d |  |
|  | BF_treat_0 | 3 | 3.54×10^-06^ | 8.97×10^-07^ | fgh |  | nd | nd |  |  |
|  | AF_1 | 3 | 2.41×10^-07*^ | 1.55×10^-07^ | a |  | nd | nd |  |  |
|  | RG_1 | 3 | 4.31×10^-06^ | 4.65×10^-07^ | hi |  | 71^*^ | 2 | c |  |
| *Chlorella variabilis N*  NC64A | BF_0 | 3 | 4.74×10^-06^ | 3.99×10^-07^ | ij | 3 | 85 | 4 | d |  |
|  | BF_treat_0 | 3 | 3.54×10^-06*^ | 6.92×10^-07^ | fgh |  | nd | nd |  |  |
|  | AF_1 | 3 | 2.69×10^-06*^ | 1.22×10^-06^ | ef |  | nd | nd |  |  |
|  | RG_1 | 3 | 1.85×10^-06*^ | 1.31×10^-07^ | e |  | 46^*^ | 14 | a |  |
| *Chlorella vulgaris*  SAG 211-11b | BF_0 | 3 | 9.01×10^-06^ | 4.84×10^-07^ | l | 1 | 86 | 9 | d |  |
|  | BF_treat_0 | 3 | 5.89×10^-06*^ | 2.65×10^-06^ | gik |  | nd | nd |  |  |
|  | AF_1 | 3 | 3.20×10^-06*^ | 9.68×10^-07^ | fg |  | nd | nd |  |  |
|  | RG_1 | 3 | 9.75×10^-06^ | 4.51×10^-06^ | kl |  | 90 | 4 | d |  |
| *Micractinium conductrix*  SAG 241.80 | BF_0 | 3 | 6.22×10^-06^ | 1.50×10^-06^ | jk | 1 | 76 | 14 | cd |  |
|  | BF_treat_0 | 3 | 4.28×10^-06*^ | 6.81×10^-07^ | gi |  | nd | nd |  |  |
|  | AF_1 | 3 | 1.79×10^-06*^ | 9.06×10^-07^ | cde |  | nd | nd |  |  |
|  | RG_1 | 3 | 6.95×10^-06^ | 1.18×10^-06^ | k |  | 60^*^ | 5 | b |  |
| **Fungus** |  |  | mol ATP g protein^-1^ |  |  |  | Oxygen consumption g DW^-1^ | | |  |
| *Aspergillus nidulans* | cold_0 | 3 | 3.88×10^-06^ | 3.71×10^-06^ | b | 1 | nd | nd |  |  |
|  | BF_0 | 3 | 4.73×10^-06^ | 7.79×10^-07^ | b |  | 249 | 140 | a |  |
|  | BF_treat_0 | 3 | 8.90×10^-08*^ | 1.19×10^-07^ | a |  | nd | nd |  |  |
|  | AF_1 | 3 | 3.71×10^-07*^ | 4.62×10^-07^ | a |  | nd | nd |  |  |
|  | RG_1 | 3 | 3.86×10^-06^ | 1.89×10^-06^ | b |  | 599^*^ | 375 | b |  |
| **Plant tissue** |  |  | mol ATP g FW^-1^ |  |  |  | Plantlet recovery | |  |  |
| *Arabidopsis thaliana* | BF_contr | 3 | 1.09×10^-08^ | 3.37×10^-09^ | b | 4 | nd | nd |  |  |
|  | BF_prep | 3 | 9.89×10^-09^ | 2.18×10^-09^ | b |  | nd | nd |  |  |
|  | BF_treat_0 | 3 | 3.47×10^-09*^ | 1.72×10^-09^ | a |  | nd | nd |  |  |
|  | AF_1 | 3 | 3.00×10^-09*^ | 1.08×10^-09^ | a |  | nd | nd |  |  |
|  | RG_1 | 3 | 2.93×10^-09*^ | 2.58×10^-09^ | a |  | nd | nd |  |  |
|  | RG_2 | 3 | 8.24×10^-09^ | 2.56×10^-09^ | b |  | nd | nd |  |  |
|  | RG_3 | 3 | 1.15×10^-08^ | 5.32×10^-09^ | b |  | nd | nd |  |  |
|  | RG_4 | 3 | 1.58×10^-08^ | 1.03×10^-08^ | bc |  | nd | nd |  |  |
|  | RG_5 | 3 | 2.93×10^-08*^ | 3.87×10^-09^ | d |  | nd | nd |  |  |
|  | RG_6 | 3 | 2.73×10^-08*^ | 7.83×10^-09^ | cd |  | nd | nd |  |  |
|  | RG_7 | 3 | 2.92×10^-08*^ | 6.76×10^-09^ | d |  | nd | nd |  |  |
|  | RG_25 | 4 |  |  |  |  | 98 | 2.8 |  |  |
| **Plant cell line** *S****.*** *tuberosum* cv. *Désiree* (DSMZ No. PC-1182) | | | mol ATP g FW^-1^ |  |  |  | Evans blue staining | |  |  |
| *Solanum tuberosum*  0 M Sorbitol | BF_0 | 6 | 9.56×10^-09^ | 6.86×10^-10^ | ij | 2/3 | 96 | 7 | d |  |
|  | BF_treat_0 | 6 | 8.25×10^-09^ | 1.30×10^-09^ | hj |  | 50^*^ | 21 | bc |  |
|  | AF_1 | 6 | 1.80×10^-10*^ | 2.41×10^-11^ | c |  | 2^*^ | 5 | a |  |
|  | RG_1 | 5 | -4.90×10^-11*^ | 1.42×10^-11^ | b |  | 8^*^ | 17 | a |  |
|  | RG_5 | 5 | -1.20×10^-10*^ | 1.13×10^-11^ | a |  | 9^*^ | 10 | a |  |
| *Solanum tuberosum*  0.3M Sorbitol | BF_treat_0 | 6 | 9.80×10^-09^ | 2.79×10^-09^ | hj | 2 | 56^*^ | 17 | c |  |
|  | AF_1 | 6 | 9.07×10^-10*^ | 2.07×10^-10^ | d |  | 6^*^ | 10 | a |  |
|  | RG_1 | 4 | 1.97×10^-10*^ | 3.10×10^-10^ | abc |  | 1^*^ | 2 | a |  |
|  | RG_5 | 6 | 9.81×10^-10*^ | 1.94×10^-09^ | abcdef |  | 8^*^ | 12 | a |  |
| *Solanum tuberosum*  0.6 M Sorbitol | BF_treat_0 | 6 | 5.97×10^-09*^ | 9.88×10^-10^ | h | 1 | 24^*^ | 19 | ac |  |
|  | AF_1 | 6 | 1.92×10^-09*^ | 3.32×10^-10^ | f |  | 5^*^ | 8 | a |  |
|  | RG_1 | 4 | 2.94×10^-09*^ | 9.54×10^-10^ | fg |  | 2^*^ | 4 | a |  |
|  | RG_5 | 6 | 9.11×10^-09^ | 6.38×10^-09^ | abcdfghj |  | 16^*^ | 16 | ab |  |
| *Solanum tuberosum*  1.2 M Sorbitol | BF_treat_0 | 6 | 5.95×10^-09*^ | 1.38×10^-09^ | h | 1 | 31^*^ | 24 | ac |  |
|  | AF_1 | 6 | 3.02×10^-09*^ | 4.46×10^-10^ | eg |  | 6^*^ | 8 | a |  |
|  | RG_1 | 5 | 6.13×10^-09^ | 2.66×10^-09^ | ghi |  | 1^*^ | 2 | a |  |
|  | RG_5 | 6 | 1.41×10^-08^ | 3.78×10^-09^ | j |  | 51^*^ | 19 | c |  |
| **Human cell line** |  |  | mol ATP viable cell^-1^ |  |  |  | Trypan blue staining | |  |  |
| *JURL-MK1* | BF_0 | 3 | 2.14×10^-11^ | 1.64×10^-11^ | a | 1 | 98 | 0 | a |  |
|  | BF_treat_0 | 3 | 1.84×10^-11^ | 1.27×10^-11^ | a |  | 98 | 0 | a |  |
|  | AF_1 | 3 | 1.45×10^-11^ | 4.35×10^-12^ | a |  | 97 | 2 | a |  |
|  | RG_1 | 3 | 1.70×10^-11^ | 2.14×10^-12^ | a |  | 99 | 1 | a |  |
