# Supplementary material for: ATP Content and Cell Viability as Indicators for Cryostress Across the Diversity of Life
Source: Front Physiol. 2018 Jul 17;9:921. doi: 10.3389/fphys.2018.00921 (PMC6056685; doi:10.3389/fphys.2018.00921)
Supplement: Supplementary file 2 [file Table_2.DOCX]

Table S2. Pairwise correlations for the association between paired samples within one organism group were tested with R (corr.test), using two-sided Spearman's rank correlation rho. Calculated are the value of the test statistic (statistic), the estimated measure of the association (estimate) and the p-value of the test, indicating the significance of the correlation (Sign.). Strains: *Pla.* *donghaensis* DSM 22276^T^, *Pla.* *halocryophilus* DSM 24743^T^, *Pla. plakortidis* DSM 23997^T^, *Psy.* *aquaticus* DSM 15339^T^, *Psy.* *cryohalolentis* DSM 17306^T^, *Psy. marincola* DSM 14160^T^, *Ch. reinhardtii* SAG 11-32b, *Chl. variabilis* ATCC 30562, *Chl. variabilis* NC64A, *Chl. vulgaris* SAG 211-11b, *M. conductrix* SAG 241.80, *Aspergillus nidulans, S. tuberosum* cv. *Désiree* (DSMZ No. PC-1182). *Pla. Planococcus, Psy. Psychrobacter, Ch. Chlamydomonas, Chl. Chlorella, M. Micractinium, S. Solanum.* via viability. ns not significant. * significant, p<0.05. ** significant p<0.01.

| **correlation** | **pair** | **estimate** | **statistic** | **p. value** | **Sign.** |
| --- | --- | --- | --- | --- | --- |
| Intracellular ATP content between bacterial strains | *Pla. donghaensis - Pla. halocryophilus* | 0.92 | 22.54 | 2.09×10 ^-05^ | ** |
|  | *Pla. donghaensis - Pla. plakortidis* | 0.77 | 66.62 | 3.60×10 ^-03^ | ** |
|  | *Pla. donghaensis - Psy. aquaticus* | 0.75 | 71.62 | 5.00×10 ^-03^ | ** |
|  | *Pla. donghaensis - Psy. cryohalolentis* | 0.92 | 22.54 | 2.09×10 ^-05^ | ** |
|  | *Pla. donghaensis - Psy. marincola* | 0.75 | 72.63 | 5.33×10 ^-03^ | ** |
|  | *Pla. halocryophilus - Pla. plakortidis* | 0.68 | 91.32 | 1.48×10 ^-02^ | * |
|  | *Pla. halocryophilus - Psy. aquaticus* | 0.83 | 48.67 | 8.38×10 ^-04^ | ** |
|  | *Pla. halocryophilus - Psy. cryohalolentis* | 0.93 | 19.07 | 9.27×10 ^-06^ | ** |
|  | *Pla. halocryophilus - Psy. marincola* | 0.80 | 58.20 | 1.93×10 ^-03^ | ** |
|  | *Pla. plakortidis - Psy. aquaticus* | 0.37 | 180.63 | 2.39×10 ^-01^ | ns |
|  | *Pla. plakortidis - Psy. cryohalolentis* | 0.55 | 127.45 | 6.14×10 ^-02^ | ns |
|  | *Pla. plakortidis - Psy. marincola* | 0.36 | 182.14 | 2.46×10 ^-01^ | ns |
|  | *Psy. aquaticus - Psy. cryohalolentis* | 0.92 | 24.08 | 2.89×10 ^-05^ | ** |
|  | *Psy. aquaticus - Psy. marincola* | 0.96 | 12.04 | 9.71×10 ^-07^ | ** |
|  | *Psy. cryohalolentis - Psy. marincola* | 0.89 | 31.11 | 9.96×10 ^-05^ | ** |
| Intracellular ATP content between algal strains | *Ch. reinhardtii - Chl. variabilis A* | 0.46 | 154.77 | 1.34×10 ^-01^ | ns |
|  | *Ch. reinhardtii - Chl. variabilis N* | 0.73 | 76.63 | 6.79×10 ^-03^ | ** |
|  | *Ch. reinhardtii - Chl. vulgaris* | 0.55 | 129.73 | 6.60×10 ^-02^ | ns |
|  | *Ch. reinhardtii - M. conductrix* | 0.49 | 145.75 | 1.06×10 ^-01^ | ns |
|  | *Chl. variabilis A - Chl. variabilis N* | -0.07 | 306.00 | 8.34×10 ^-01^ | ns |
|  | *Chl. variabilis A - Chl. vulgaris* | 0.85 | 44.00 | 9.70×10 ^-04^ | ** |
|  | *Chl. variabilis A - M. conductrix* | 0.89 | 32.00 | 9.17×10 ^-05^ | ** |
|  | *Chl. variabilis N - Chl. vulgaris* | -0.02 | 292.00 | 9.56×10 ^-01^ | ns |
|  | *Chl. variabilis N - M. conductrix* | -0.03 | 294.00 | 9.39×10 ^-01^ | ns |
|  | *Chl. vulgaris - M. conductrix* | 0.92 | 24.00 | 0.00×10 ^+00^ | ** |
| Intracellular ATP content between different sorbitol treatments of *Solanum tuberosum* | *S. tuberosum* 0 M - *S. tuberosum* 0.3 M | 0.83 | 544.17 | 6.57×10 ^-08^ | ** |
|  | *S. tuberosum* 0 M - *S. tuberosum* 0.6 M | 0.36 | 2097.82 | 6.54×10 ^-02^ | ns |
|  | *S. tuberosum* 0 M - *S. tuberosum* 1.2 M | -0.14 | 4148.20 | 4.93×10 ^-01^ | ns |
|  | *S. tuberosum* 0.3 M - *S. tuberosum* 0.6 M | 0.33 | 2178.83 | 8.77×10 ^-02^ | ns |
|  | *S. tuberosum* 0.3 M - *S. tuberosum* 1.2 M | -0.03 | 3773.05 | 8.69×10 ^-01^ | ns |
|  | *S. tuberosum* 0.6 M - *S. tuberosum* 1.2 M | 0.72 | 1018.28 | 1.48×10 ^-05^ | ** |
| Intracellular ATP content and viability | All microorganisms before freezing | 0.63 | 106 | 3.2×10 ^-02^ | * |
|  | All microorganisms after regrowth | 0.57 | 124 | 5.9×10 ^-02^ | ns |
| Intracellular ATP content and viability | *Aspergillus nidulans* | -0.37 | 48.00 | 4.97×10 ^-01^ | ns |
|  | *Pla. donghaensis* | 0.24 | 216.00 | 4.44×10 ^-01^ | ns |
|  | *Pla. halocryophilus* | -0.09 | 312.05 | 7.78×10 ^-01^ | ns |
|  | *Pla. plakortidis* | -0.07 | 305.03 | 8.37×10 ^-01^ | ns |
|  | *Psy. aquaticus* | -0.01 | 288.00 | 9.83×10 ^-01^ | ns |
|  | *Psy. cryohalolentis* | 0.17 | 236.91 | 5.94×10 ^-01^ | ns |
|  | *Psy. marincola* | -0.16 | 331.08 | 6.25×10 ^-01^ | ns |
|  | *Ch. reinhardtii* | 0.89 | 3.74 | 1.65×10 ^-02^ | * |
|  | *Chl. variabilis A* | -0.31 | 46.00 | 5.4×10 ^-01^ | ns |
|  | *Chl. variabilis N* | 0.83 | 6.00 | 4.2×10 ^-02^ | * |
|  | *Chl. vulgaris* | -0.14 | 40.00 | 8.03×10 ^-01^ | ns |
|  | *M. conductrix* | -0.54 | 54.00 | 2.97×10 ^-01^ | ns |
|  | *S. tuberosum* 0.0 M Sorbitol | 0.68 | 430.61 | 1.06×10 ^-03^ | ** |
|  | *S. tuberosum* 0.3 M Sorbitol | 0.64 | 199.39 | 9.58×10 ^-03^ | ** |
|  | *S. tuberosum* 0.6 M Sorbitol | 0.70 | 170.65 | 4.01×10 ^-03^ | ** |
|  | *S. tuberosum* 1.2 M Sorbitol | 0.53 | 320.74 | 3.54×10 ^-02^ | * |
