# Supplementary material for: ATP Content and Cell Viability as Indicators for Cryostress Across the Diversity of Life
Source: Front Physiol. 2018 Jul 17;9:921. doi: 10.3389/fphys.2018.00921 (PMC6056685; doi:10.3389/fphys.2018.00921)

Supplementary Figure 1. Workflow of the cryostress experiments conducted for bacteria, algae, a fungus, plant tissue, a plant cell line, and a human cell line. Cultivation was done under optimal conditions before freezing (BF). Steps of the cryostress experiments comprised treatment with cryoprotectants (BF_treat), freezing and thawing (AF). During regeneration, cells were regrown under optimal conditions. TSY, tryptic soy yeast medium; MB, Marine Broth; ESF1, basal medium with beef extract; TAP, Tris-acetate-phosphate medium; AMM, *Aspergillus* minimal medium; MS, Murashige and Skoog medium containing 0.1 M sucrose; BF_contr, control (wildtype seedlings); BF_prep, prepared (freshly excised shoot tips); DMSO, dimethylsulfoxid; PVS2, plant vitrification solution; FBS, fetal bovine serum; LN_2, liquid nitrogen; OD, optical density; TCN, total cell number; FDA, fluorescein diacetate; RPMI, RPMI 1640 Medium developed at Roswell Park Memorial Institute.

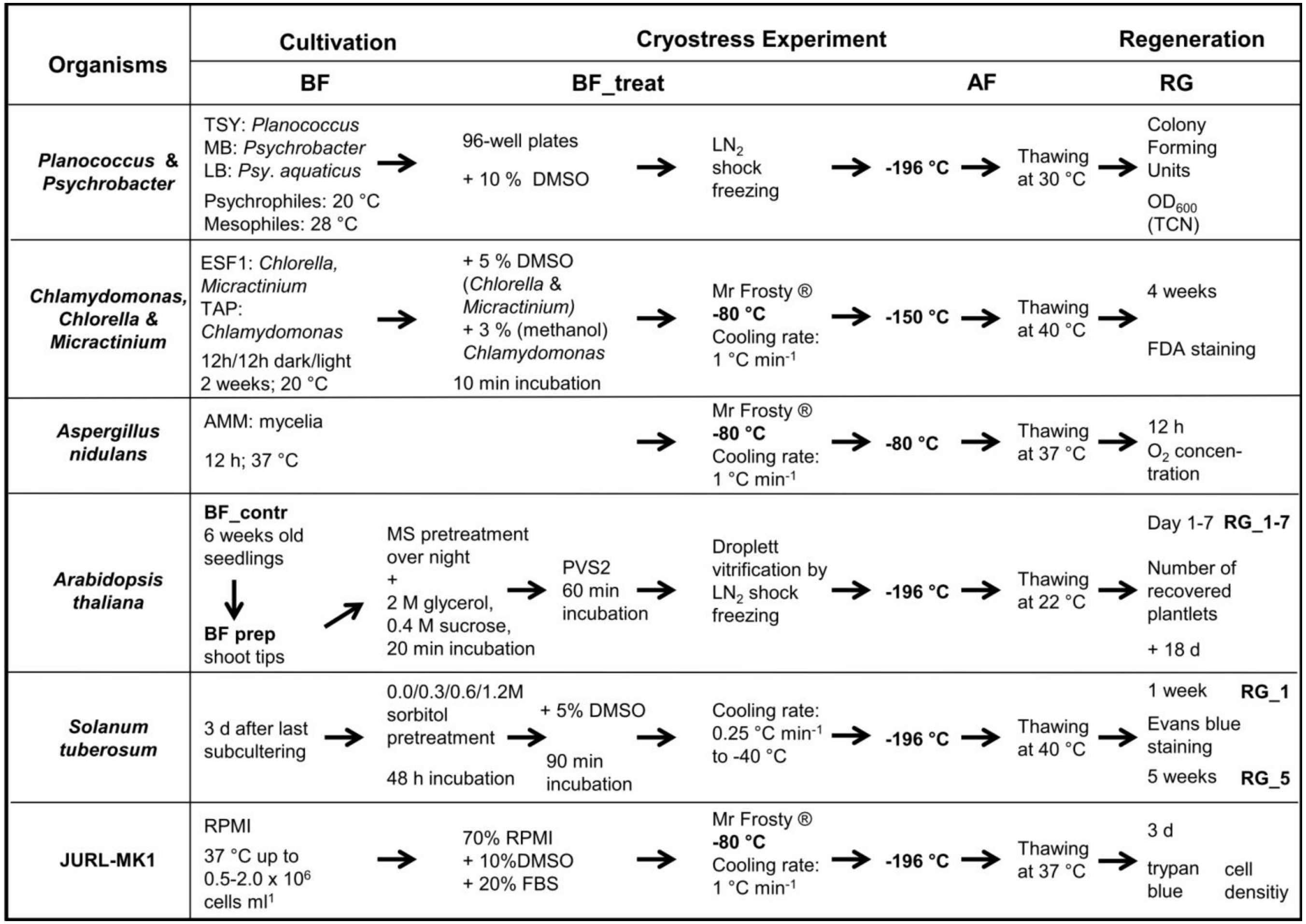

Supplement: Supplementary file 3 [file Image_1.PDF]
