# Supplementary material for: ATP Content and Cell Viability as Indicators for Cryostress Across the Diversity of Life
Source: Front Physiol. 2018 Jul 17;9:921. doi: 10.3389/fphys.2018.00921 (PMC6056685; doi:10.3389/fphys.2018.00921)

Fig. S2. General patterns of the changes in intracellular ATP content observed during cryostress experiments. In order to verify and classify the distinct patterns in ATP content across the different model organisms under cryostress, the pattern determined for each strains or organism was fitted to different shapes employing the R package dtwclust (Fig. 3). The GAK algorithm performed best to describe the observed trends in cellular ATP. This analysis resulted in clusters of strains or organisms that could be assigned to four different shapes. BF, before freezing; BF_treat, after treatment with cryoprotectant; AF, after freezing; RG, after the regrowth phase.

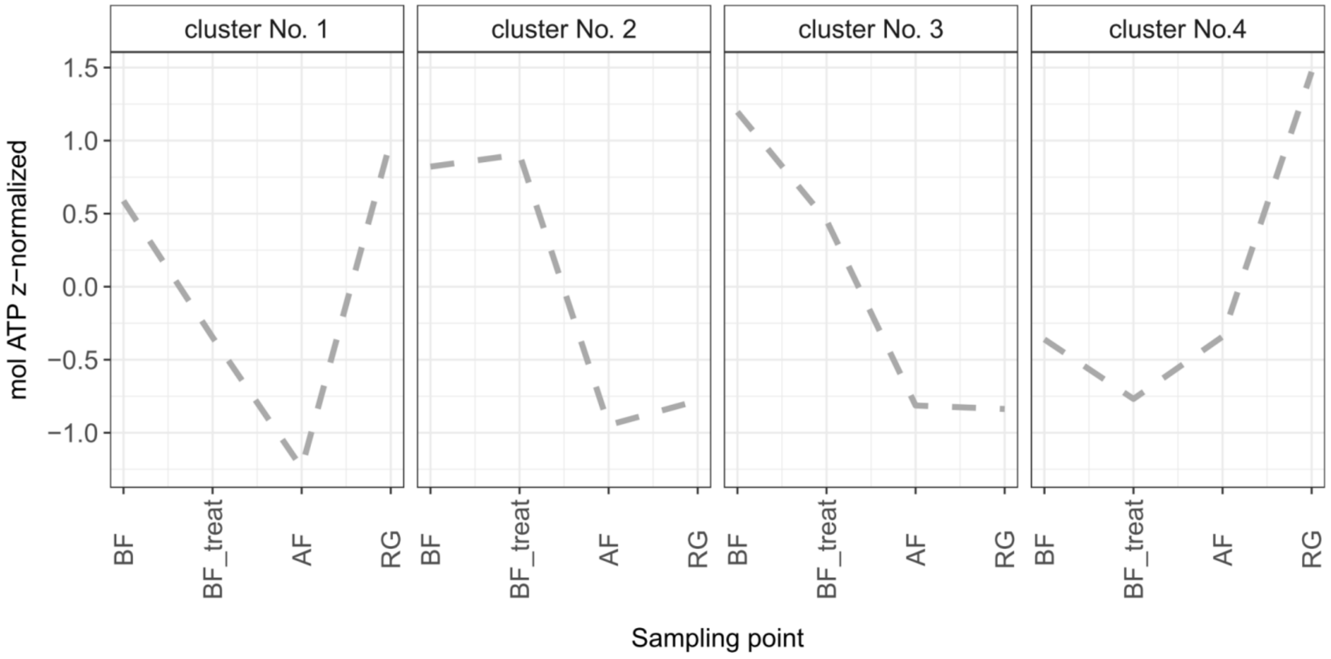

Supplement: Supplementary file 4 [file Image_2.PDF]
