# Supplementary material for: ATP Content and Cell Viability as Indicators for Cryostress Across the Diversity of Life
Source: Front Physiol. 2018 Jul 17;9:921. doi: 10.3389/fphys.2018.00921 (PMC6056685; doi:10.3389/fphys.2018.00921)

Supplementary Figure 3. Regeneration of plant tissue and cell lines. A. Developmental stages of *Arabidopsis thaliana* shoot tips during regeneration. **B.** Recovery of Col-0 wildtype plantlets 25 days after cryopreservation. Scale bar: 1 mm. **C.** The success of plant-survival in cryopreservation strongly depends on (osmotic) pre-acclimatization. Calli of *Solanum tuberosum* cv. Désiree after 5 weeks of regrowth (RG_5) are depicted. Viability increased with increasing molarity of the sorbitol pretreatment (0.0 < 0.3 < 0.6 < 1.2 M).

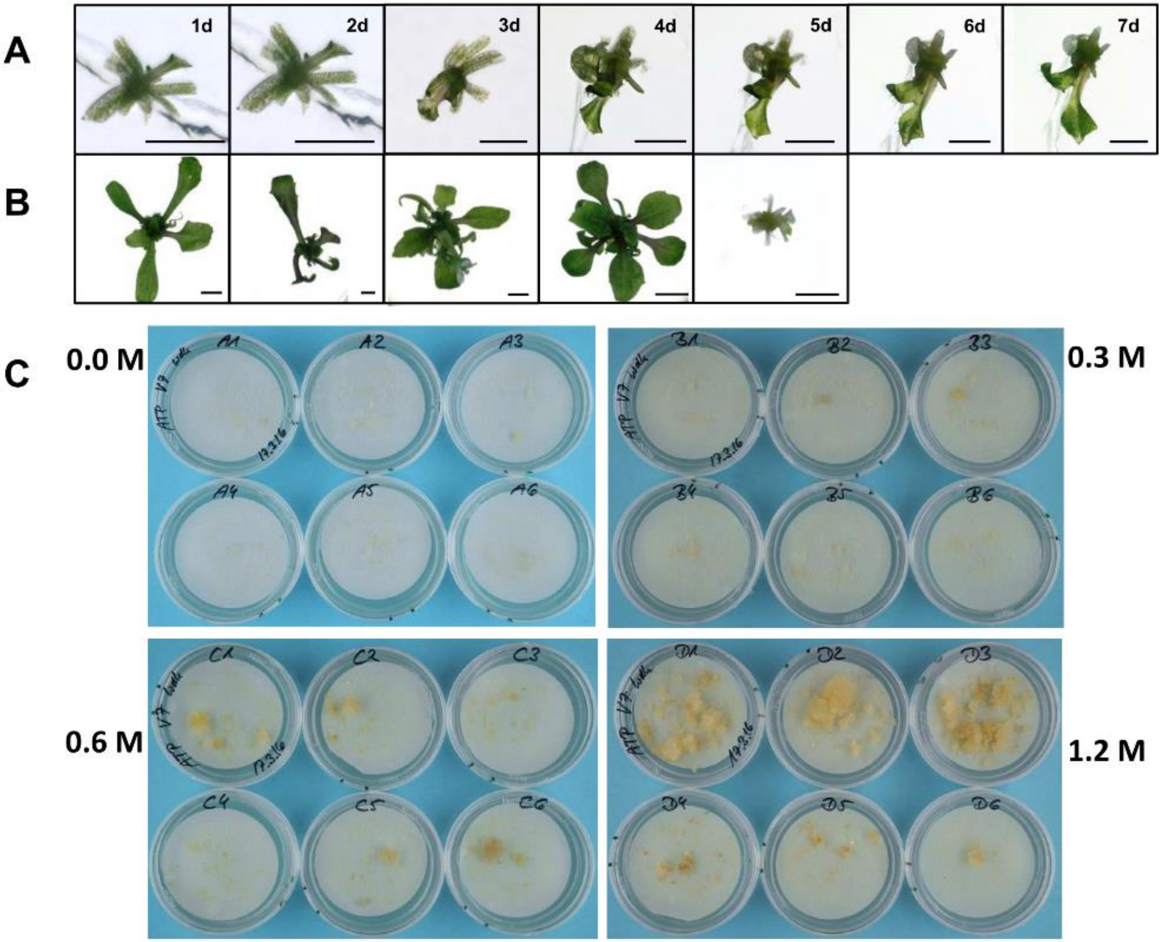

Supplement: Supplementary file 5 [file Image_3.PDF]
